# Prevalence and clinical correlates of *Schistosoma mansoni* co-infection among malaria infected patients, Northwest Ethiopia

**DOI:** 10.1186/s13104-015-1468-2

**Published:** 2015-09-28

**Authors:** Sisay Getie, Yitayih Wondimeneh, Gebeyaw Getnet, Meseret Workineh, Ligabaw Worku, Afework Kassu, Beyene Moges

**Affiliations:** Department of Medical Parasitology, School of Medicine and Health Sciences, College of Medicine and Health Sciences, University of Gondar, Gondar, Ethiopia; Department of Immunology and Molecular Biology, School of Biomedical and Laboratory Sciences, College of Medicine and Health Sciences, University of Gondar, Gondar, Ethiopia; Department of Medical Microbiology, School of Biomedical and Laboratory Sciences, College of Medicine and Health Sciences, University of Gondar, Gondar, Ethiopia

**Keywords:** Malaria, *Schistosoma mansoni*, Co-infection, Ethiopia

## Abstract

**Background:**

In Ethiopia, where malaria and schistosomiasis are co-endemic, co-infections are expected to be high. However, data about the prevalence of malaria-schistosomiasis co-infection and their clinical correlation is lacking. Therefore, the aim of this study was to assess prevalence of *Schistosoma mansoni* co-infection and associated clinical correlates in malaria patients.

**Methods:**

A cross-sectional study was conducted in 2013 at Chwahit Health Center, in northwest Ethiopia. Blood film positive malaria patients (N = 205) were recruited for the study. Clinical, parasitological, hematological, and biochemical parameters were assessed from every study participant. Stool samples were also collected and processed with Kato-Katz technique to diagnose and classify intensity of *Schistosoma mansoni*.

**Results:**

The prevalence of *Schistosoma mansoni* and malaria co-infection was 19.5 %. The age group of 16–20 years old was significantly associated with co-infection. Co-infected patients with a moderate-heavy egg burden of *Schistosoma mansoni* had significantly high mean *Plasmodium* parasitemia. On the other hand, age group of 6–10 years old and moderate-heavy *Schistosoma mansoni* co-infection were significantly associated with severe malaria.

**Conclusions:**

Prevalence of malaria and *Schistosoma mansoni* co-infection in the study area was considerably high. Severity of malaria and parasitemia of *Plasmodium* were associated with certain age groups and intensity of concurrent *Schistosoma mansoni*. Further study is needed to explore the underlying mechanisms of interaction between malaria and *Schistosoma mansoni*.

## Background

Malaria is a complex and deadly parasitic disease caused by genus *Plasmodium.Plasmodium falciparum* (*P. falciparum*) and *Plasmodium vivax* (*P. vivax*) are the predominant species globally attributing to the majority of disease burden. According to *World Malaria Report* 2013, there were an estimated 207 million episodes and 627,000 deaths due to malaria in 2012, of which approximately 80 % of the episodes and 90 % of the deaths were in the Africa region. Approximately, 77 % of malaria deaths globally occurred among children under 5 years [1].

Malaria is a major public health burden in Ethiopia, where its transmission and burden varies with altitude, degree of urbanization, and use of insecticides [2]. A recent malaria trend analysis in northwest Ethiopia revealed 40 % prevalence; the only species to cause the disease, as confirmed microscopically, were *P. falciparum* (75 %) and *P. vivax* (25 %) [3].

In tropical African countries, epidemiological concurrence and co-infection between malaria and helminthiasis is common [4–6]. For example, in Tanzania the prevalence of malaria-schistosomiasis co-infection was reported to be 42 % [4]. In Ethiopia, the prevalence of malaria-schistosomiasis co-infection reported to be 15 % and caused high prevalence of anemia, as compared to those infected only with malaria [6].

Earlier studies indicated a controversial interaction of helminthic co-infection with malaria [7, 8]. Intestinal schistosomiasis plays an antagonistic role against malaria, but the egg intensity of *Schistosoma mansoni* (*S. mansoni*) and the age of infected individuals could determine the type of interaction [9, 10]. Although most studies were conducted on animal models, they reported that *S. mansoni* co-infection contributes to severe malaria presentation. They revealed that *S. mansoni* co-infection resulted in high *Plasmodium* parasitemia and increased susceptibility of infected mouse models to mortality [11–14]. In contrast, others illustrated that *S. mansoni* co-infection contributed to low *Plasmodium* parasitemia and inhibited cerebral malaria [15–17]. However, these studies suggested that the type of animal model and *Plasmodium* parasite used significantly affects the impact of *S. mansoni* co-infection with malaria.

On the other hand, a study among humans revealed the association between heavy *Plasmodium* parasitemia and heavy intensity schistosomiasis co-infection [18]. In order to put better clinical management and control of malaria, especially in schistosomiasis co-endemic areas, information on the prevalence and clinical outcomes of schistosomiasis co-infection with malaria is needed. Therefore, this study was aimed to assess the prevalence of intestinal *S. mansoni* and malaria co-infection and associated clinical correlates among patients in northwest Ethiopia.

## Methods

### Study area

The study was conducted at Chwahit Health Center, Northwest Ethiopia. Chwahit is located at 12°20ʹ4ʺN and 37°13ʹ35ʺE and at an elevation of 1837 m above sea level. There was one health center providing health care for about 30,000 people in Chwahit and its surrounding. As reported by the District Health Bureau, malaria (due to *P. falciparum* and *P. vivax*) and schistosomiasis (due to *S. mansoni*) are common in the study area.

### Study design, period and participants

A cross-sectional study was conducted from February to May 2013 among microscopically confirmed malaria patients attending Chwahit Health Center.

### Inclusion criteria

Microscopically confirmed malaria patients who provided both blood and stool samples were enrolled in the study.

### Exclusion criteria

Patients who attended antiretroviral therapy and antenatal outpatient departments, and patients who had confirmed chronic diseases and concurrent intestinal helminths other than *S. mansoni* were excluded from participation.

### Sample size and sampling techniques

Single population proportion formula was used. Assuming 15 % of malaria and *S. mansoni* co-infection [6], 95 % level of significance, 5 % precision and 5 % non response rate, a total of 205 study participants were recruited using a random sampling technique.

### Data collection procedures

#### Demographic and clinical data

An interview-based questionnaire was used to collect demographic data, and clinical data was collected during physical examination at the outpatients’ department of the Health Centre. Severe malaria, in this study, was defined as: *P. falciparum* positive patients who presented with either circulatory collapse (a systolic pressure of less than 80 mmHg in adults and <50 mmHg in children), severe anemia less than 5 mg/dl, hypoglycemia less than 40 mg/dl, or hyper-parasitemia (≥100,000 parasites/μl of whole blood) [19].

### Microscopic determination of *Plasmodium* parasitemia

Two drops of venous blood were placed separately on a microscopic glass slide. Thick and thin blood films were prepared and air dried. Thin blood films were fixed with absolute methanol and both films were stained with 10 % Geimsa working solution for 10 min. Then both thin and thick blood films were read by an experienced malaria microscopist with a 100× objective lens and 100 microscopic fields examined to rule out the absence of the malaria parasite. Malaria parasitemia was determined in thick blood films along with 200 white blood cells.

### Determination of hematological parameters

Three milliliters venous blood was collected with tris-potassium ethylene diamine tetra acetic acid (EDTA) anti-coagulated tube. Blood was analysed by Cell Dyne 1800 (Abbot Hematology, IL, USA) for the determination of total white blood cells, granulocytes, lymphocytes, mixed cells, red blood cells, platelets, and hemoglobulin level.

### Serum biochemical analysis

Three milliliters venous blood was collected with non-anticoagulated tube and allowed to clot at bench top. Then, blood centrifuged at 3000 revolution per minute for 4 min and serum was aliquoted. Then, serum was analysed by HumaStar chemistry analyzer (Human Diagnostics, USA) for serum level of serum glutamate pyruvate transaminase (SGPT), serum glutamate oxaloacetate transaminase (SGOT), total protein, and glucose.

### Microscopic diagnosis of *Schistosoma mansoni*

One gram of fresh stool sample was collected from every study participant. Stool samples were processed with Kato-Katz technique using standardized template measuring 41.75 mg of stool to prepare smear for detection and count of *S. mansoni* ova. The egg load of *S. mansoni* from stool was classified as light [1–100 egg/gram (epg)], medium (101–400 epg), or heavy (>400 epg) [20].

### Statistical analysis

Data entered into computer data base using Epi Info 3.5.3. Cleared data was transferred to SPSS version 20 for statistical analysis using logistic regression, independent samples *t* test and one way ANOVA. Odds ratio (OR) with 95 % CI was used to determine strength of association between variables. Statistically significance was considered at 95 % level of confidence and P value less than 0.05.

### Ethical considerations

The study protocol was reviewed and approved by School of Biomedical and Laboratory Sciences Research and Ethics Committee, University of Gondar, Gondar, Ethiopia. Permission was obtained from Dembia Health Bureau and Chwahit Health Center to conduct the study. Informed consent and assent were obtained from all participants and/or their guardians after being briefed on the risks and benefits of the study.

## Results

### Demographic and clinical characteristics of the study participants

A total of 205 malaria positive study participants, 63.4 % (130/205) males and 36.6 % (75/205) females, were included. The mean (SD) age of study participants was 25 (12) years. The prevalence of *P. falciparum* and *P. vivax* among the study participants was 71.7 % (147/205) and 25.9 % (53/205), respectively. The remaining 2.4 % (5/205) had mixed infections. Fifty six percent (115/205) of the study participants presented with fever. Hypotension was found only among 2.9 % (6/205) of the participants. Severe malaria was observed in 25 % (38/152) of the study participants, while hyper-parasitemia was identified in only 3.9 % (6/152) of the study participants harboring *P. falciparum.*

### Prevalence of malaria and *S. mansoni* co-infection

In this study, the prevalence of *S. mansoni* and malaria co-infection was 19.5 % (40/205). Eighty percent of the *S. mansoni* co-infections were observed in participants with *P. falciparum*, while the remaining 20 % were within those with *P. vivax* infection. A higher percentage of *S. mansoni* co-infection [32.5 % (13/40)] was observed within the age group of 16–20 years old (p < 0.05) (Table 1).

**Table 1:** Logistic regression for risk of *S. mansoni* co-infection among study participants at Chwahit Health Center, Northwest Ethiopia, 2013

Variables	Frequency of *S. mansoni* co-infection		β (95 % CI)
	*S. mansoni* co-infected (%)	Malaria only infected (%)	
Sex			
Male	28 (21.5)	102 (78.5)	1
Female	12 (16.0)	63 (84.0)	0.686 (0.31–1.516)
Age			
6–10	5 (23.8)	16 (76.2)	2.494 (0.661–9.41)
11–15	5 (18.2)	21 (80.8)	1.958 (0.52–7.37)
16–20	13 (26.0)	37 (74.0)	3.04 (1.04–8.94)*
21–25	6 (20.0)	24 (80.0)	2.22 (0.63–7.83)
26–30	5 (20.0)	20 (80.0)	1.97 (0.53–7.39)
30+	6 (11.3)	47 (88.7)	1.00
Residence			
Urban	6 (13.3)	39 (86.7)	1.00
Rural	34 (21.4)	126 (78.6)	1.57 (0.58–4.2)
Living nearby swampy area			
Yes	22 (22.7)	75 (77.3)	1.54 (0.73–3.26)
No	18 (16.7)	90 (83.3)	1.00

*β* coefficient of regression, *CI* confidence interval

* Statistically significant with p value less than 0.05

### Clinical correlates of malaria and *S. mansoni* co-infection

In this study, there was a higher frequency of fever among *S. mansoni* co-infected participants than malaria only infected ones (χ^2^ = 5.43, P < 0.022). A higher frequency of severe anemia was also observed among *S. mansoni* co-infected participants (35.0 %) than malaria only infected ones (25.5 %) (χ^2^ = 7.54, P < 0.006).

In addition, mean differences in hematological and biochemical parameters were observed among *S. mansoni* co-infected and malaria only infected participants. However, a significant difference was observed only in mean red blood cell count (4.42 ± 0.11 vs. 4.64 ± 0.04, p < 0.033) and hemoglobulin level (12.48 ± 0.38 vs. 13.38 ± 0.13, p < 0.006), respectively (Tables 2, 3).

**Table 2:** Independent t test for blood cells count, mean difference between *S. mansoni* co-infected and malaria only infected participants at Chwahit Health Center, Northwest Ethiopia, 2013

Parameters	Malaria only infected	*S. mansoni* co-infected	p value
Total white blood cell (×10^3^/μl)	5.81 ± 0.15	5.44 ± 0.24	<0.285
Granulocyte (×10^3^/μl)	3.44 ± 0.12	3.25 ± 0.23	<0.489
Lymphocyte (×10^3^/μl)	1.71 ± 0.06	1.6 ± 0.08	<0.462
Mixed cells (×10^3^/μl)	0.76 ± 0.045	0.59 ± 0.21	<0.062
Red blood cells (×10^6^/μl)	4.64 ± 0.04	4.42 ± 0.11	<0.033
Haemoglobulin (g/dl)	13.38 ± 0.13	12.48 ± 0.38	<0.006
Platelet (×10^3^/μl)	182.4 ± 4.5	190.3 ± 7.8	<0.42

**Table 3:** Independent t test for SGOT, SGPT, glucose, total protein and parasitaemia mean difference between *S. mansoni* co-infected and malaria only infected participants at Chwahit Health Center, Northwest Ethiopia, 2013

Parameters	Malaria only infected	*S. mansoni* co-infected	p value
SGOT (IU/L)	34.00 ± 2.02	32.06 ± 1.6	<0.64
SGPT (IU/L)	33.5 ± 3.06	30 ± 2.52	<0.58
Glucose (mg/dl)	72.13 ± 1.7	67.92 ± 2.2	<0.24
Total protein (g/dl)	5.78 ± 0.08	5.73 ± 0.16	<0.81
Parasitaemia (parasite/μl)	4053 ± 206	3917 ± 393	<0.77

There was a mean difference in malaria parasitemia among malaria only infected, light and moderate-heavy *S. mansoni* co-infected participants (F = 3.43, p < 0.034). In the Post Hoc analysis, this difference persisted only among light and moderate-heavy *S. mansoni* co-infected participants (3224 vs. 5537 parasite/μl, p < 0.027).

In this study, severe malaria was identified in 18.5 % (38/205) of the study participants. A higher frequency of severe malaria was observed among males (65.8 %) than females (34.2 %). Patients in the age group of 6–10 years old were 6.8 times more likely to experience severe malaria (p < 0.05). Twenty-six percent of the study participants with severe malaria had *S. mansoni* co-infection. Moderate-heavy intensity of *S. mansoni* was associated with severe malaria (p < 0.05) (Table 4).

**Table 4:** Logistic regression for severe malaria among study participants diagnosed with *Plasmodium falciparum* at Chwahit Health Center, Northwest Ethiopia, 2013

Variables	Frequency of severe malaria		β (95 % CI)
	Severe malaria (%)	Mild malaria (%)	
Sex			
Male	25 (27.5)	66 (72.5)	1.00
Female	13 (21.3)	48 (78.7)	1.64 (0.68–3.99)
Age			
6–10	9 (47.4)	10 (52.6)	6.823 (1.841–25.290)**
11–15	9 (40.9)	13 (59.1)	3.464 (0.927–12.949)
16–20	4 (13.3)	26 (86.7)	1.052 (0.277–4.000)
21–25	6 (28.6)	15 (71.4)	1.528 (0.355–6.570)
26–30	3 (15.8)	16 (84.2)	1.038 (0.205–5.258)
30+	7 (17.1)	34 (82.9)	1.00
Residence			
Urban	6 (21.4)	22 (78.6)	1.00
Rural	32 (25.8)	92 (74.2)	0.775 (0.260–2.314)
*S. mansoni* egg load			
No egg	28 (23.3)	92 (76.7)	1.00
Low intensity	3 (13.0)	20 (87.0)	0.380 (0.093–1.558)
Moderate-heavy intensity	7 (77.8)	2 (22.2)	15.581 (2.717–89.365)**

*β* coefficient of regression, *CI* confidence interval

** Statistically significant with p value less than 0.05

## Discussion

Malaria and schistosomiasis co-exist in sub-Saharan African countries [4–6, 21]. In the present study, the overall prevalence of *S. mansoni* co-infection with malaria was 19.5 %. This prevalence was lower compared to a recent study carried out in southern Ethiopia, 22.6 % [21], and Tanzania 42 % [5]. However, it was higher than another study conducted in southern Ethiopia, 15 % [6]. A cohort study in Senegal also reported a high co-infection between malaria and schistosomiasis [22]. The difference can be explained by differences in water contact and microgeographical areas which could affect the co-infection between malaria and schistosomiasis [23, 24].

In this study, higher *S. mansoni* co-infection was observed among males and within the age group of 16–20 years old. In an earlier study from Kenya, children were 9.3 times more likely to be co-infected with schistosomiasis and malaria than adults [18]. Additionally, studies from Sudan and Swaziland revealed that socio-cultural factors could lead higher exposure of males for schistosomiasis [25–27].

In the current study, a higher frequency of *S. mansoni* co-infected participants presented with fever than malaria only infected ones. Although each case of malaria and schistosomiasis could lead to fever presentation [28, 29], schistosomiasis co-infection with malaria suggested to upregulate production of inflammatory markers that aggravate fever [30].

In this study, although significant association was found only in red blood cell count and hemoglobulin level, hematological parameters were found to be influenced by *S. mansoni* co-infection. In an earlier animal model study, schistosomiasis co-infection prolonged lower density malaria parasitemia time and caused anemia as compared to the malaria only infected group [31].

However, *P. falciparum* and *P. vivax* were reported to decrease hemoglobulin level, white blood cells, red blood cells and platelet counts [32]. Schistosomiasis could also result in low hemoglobulin level and lymphocyte count, but increased total white blood cell, neutrophils, eosinophiles and monocytes count [33, 34]. Therefore, concurrent schistosomiasis with malaria could lead to high co-morbidity associated with hematological parameters.

In this study, high mean *Plasmodium* parasitemia was observed more frequently among malaria only infected participants than *S. mansoni* co-infected participants. In an earlier study, reduced *Plasmodium* parasitemia was reported to be associated with schistosomiasis co-infection [10]. However, this contradicted a result from southern Ethiopia, where schistosomiasis co-infected malaria patients were 3.65 times more likely to be *Plasmodium* dense than malaria only positives [6]. The difference could be explained with variation in the intensity of schistosomiasis co-infection, which would affect *Plasmodium* parasitemia.

Higher mean *Plasmodium* parasitemia was more frequently observed among moderate-heavy *S. mansoni* co-infected participants than light co-infected ones. Therefore, schistosomiasis co-infection could affect *Plasmodium* parasitemia, depending on the intensity of the ova. Previous studies from Mali and Senegal reported that light schistosomiasis intensity is correlated with a decrease in *Plasmodium* parasitemia [9, 35].

In the current study, the age group of 6–10 years old and moderate-heavy intensity *S. mansoni* co-infection were identified as the determining factors for severe malaria. In earlier studies, children co-infected with schistosomiasis and heavy intensity of schistosomiasis were at risk of developing severe malaria [24, 30, 35]. This could be explained by the high production of interferon gamma (IFN-γ) in children and resulted in higher malaria parasitemia that could lead to severe malaria [30]. In the present study, 66.7 % of the study participants who presented with hyper-parasitemia were children.

## Conclusions

Prevalence of malaria and *S. mansoni* in the study area was considerably high. Severity of malaria and parasitemia of *Plasmodium* were associated with certain age groups and intensity of concurrent intestinal schistosomiasis. Further study is needed to explore the underlying mechanisms of interaction between malaria and *S. mansoni*.

## Abbreviations

EDTA: tris-potassium ethylene diamine tetra acetic acid
epg: egg per gram
IFN-γ: interferon gamma
SGOT: Serum glutamate oxaloacetate transaminase
SGPT: serum glutamate pyruvate transaminase
